# Evaluation of E-Learning Experience among Health and Allied Health Professions Students during the COVID-19 Pandemic in Slovenia: An Instrument Development and Validation Study

**DOI:** 10.3390/ijerph19084777

**Published:** 2022-04-14

**Authors:** Mirko Prosen, Igor Karnjuš, Sabina Ličen

**Affiliations:** Department of Nursing, Faculty of Health Sciences, University of Primorska, Polje 42, 6310 Izola, Slovenia; igor.karnjus@fvz.upr.si (I.K.); sabina.licen@fvz.upr.si (S.L.)

**Keywords:** distance education, evaluation, instrument development, e-learning environments, information and communication technology, higher education

## Abstract

COVID-19 had an impact on everyday life, especially during the lockdown. This also impacted higher education, leading to a sudden and complete shift to online e-learning. The purpose of this study was to develop, validate, and test a measurement tool suitable for evaluating students’ e-learning experience among health and allied health professions students. The convenience sample consisted of 342 students. A validation of the instrument E-learning Experience Evaluation Scale (3E-Scale) was conducted before the study began. Factor structure, reliability, content, and face validity were assessed. Confirmatory factor analyses revealed a four-factor structure of the scale that explained 61% of the total variance. The overall scale demonstrated a high level of reliability and appears to be a reliable measurement tool. The results show that there are statistically significant differences between female and male students (*p* < 0.05). In addition, nursing and dietetics students perceive more barriers related to the open-source learning management system than other students (*p* < 0.05). Positive learning experiences contribute to greater learning satisfaction and, consequently, greater learning engagement. E-learning content design should be aligned with teaching pedagogy and learning outcomes. Future studies should also address the negative consequences of e-learning experiences.

## 1. Introduction

Over the past three decades, e-learning has come to be known by a variety of names, with “e-learning” apparently having caught on the most. E-learning is an umbrella term that encompasses various concepts and learning-related technologies such as distance, digital, electronic, online, and mobile learning [1,2,3]. While it includes and has interdisciplinary links with other disciplines and industries, e.g., computer engineering, information technology, design, and media studies, e-learning is basically a discipline of education [3,4]. While e-learning can effectively increase learners’ knowledge and knowledge-related skills through a range of activities, it must be emphasised that e-learning is not superior to the traditional approaches. It is imperative that it be used with a solid understanding of learners’ needs [3], which makes the process of education more student-centred, creative, and flexible [5,6].

Despite numerous definitions, e-learning is mostly defined as a form of online learning that uses ICT to present and distribute content as well as for interpersonal communication and interaction. In this definition, three elements distinguish e-learning from the traditional educational approaches: asynchronous delivery (absence of the time dimension), decentralisation (absence of the space dimension), and electronically mediated interaction or communication [7,8,9,10]. In this respect, the absence of space and time in e-learning significantly impacts the teacher–learner interaction, at least compared to traditional teaching forms. E-learning creates an entirely new social environment with the consequent need to develop new social skills. It is precisely this altered form of communication and interaction that in turn influences other elements of learning and teaching and calls for a new approach on the part of the teacher [5,8,11]. Although many types of e-learning are known, we can basically classify e- or online learning into synchronous, asynchronous, blended, massive online open courses, and open schedule online courses [4]. 

E-learning gives several advantages to both teacher and learners, yet it also comes with certain disadvantages. Among the advantages, research highlights the lack of dependence on time and space, the greater individualisation of learning materials inside an innovative and interactive environment, cost effectiveness in terms of labour and materials costs, flexibility and transferability, a higher degree of cooperation, consistency in delivery together with a bigger possibility of being involved in education, etc. [1,4,8,9,12,13]. The shortcomings of e-learning include the lack of spontaneity in interpersonal interaction and communication, which can negatively affect the social component of learning as well as the teacher’s ability to teach, teacher preparation, greater responsibility, and self-discipline to maintain the motivation to learn, lack of direct support from the teacher, limited technical skills, disruptions in the home or learning environment, and inadequate equipment or poor Internet connectivity [1,8,9,14].

As the need and level of education increases, so does the need to develop e-learning methods. This makes monitoring the quality of e-learning and its components extremely important for developing appropriate methods, e-learning, and embedded content; however, the evaluation of e-learning should not only consider the technologies that contribute to the e-learning environment’s design, but a holistic evaluation of the e-learning environment itself [2]. Although evaluating the e-learning tools themselves remains important, there is growing awareness of the additional need to evaluate the ways in which these tools make the e-learning environment more effective. Despite numerous attempts to introduce e-learning into higher education, little is currently known about how to approach the evaluation of e-learning [1,15,16], because few studies have specifically evaluated the e-learning experiences of health professions students [17]. Moreover, the lack of relevant feedback prevents further development of the field, which is certain to become the main mode of teaching and learning in the future. E-learning can enable students to satisfy their educational needs through various digital technologies and therefore help them build their knowledge through access to various didactic materials and through different learning and teaching methods [13,18]. Despite certain shortcomings of e-learning, it must be emphasised that e-learning is much more than the mere repetition of what was previously thought and learned in a classroom. Designing an e-learning programme or course based on pedagogical principles that take into account, among other things, student experience and satisfaction is highly motivating to students [19,20]. Positive learning experience contributes to greater learning satisfaction and, consequently, greater learning engagement. Learning satisfaction is not only an important indicator of students’ enjoyment with study, but also an important evaluation component in assessing learning effectiveness [17].

Compared to other fields of education, health professions education has traditionally been delivered face-to-face in either educational or clinical settings, as the development of personal and technical skills is an important part of the education of health professions students. However, the COVID-19 pandemic had such an impact on the learning environment that e-learning became a necessity rather than a choice in health education. As a result of these efforts, educators began searching for optimal ways to find appropriate approaches to effectively deliver course content online [11,12,19,21]. Studies [10,21,22,23] conducted to date among health professions students indicate that e-learning was well received, especially in terms of time management and performance, but less so in terms of content understanding and further explanation, methodology, technical, and behavioural challenges encountered due to the long period without face-to-face interaction. In a sample of nursing students, Warshawski [13] found that despite the acknowledged positive effects of e-learning, first-year students experienced increased workloads, difficulty managing and understanding study materials, and a lack of learning interaction. Consequently, perceptions of difficulty decreased their academic self-efficacy, which is recognised as an important motivator for academic success.

The aim of this study was to develop, validate, and test an instrument to assess the e-learning experiences of health and allied health professions students and to measure e-learning experience at a time when the need for and use of e-learning was the predominant mode of learning.

## 2. Materials and Methods

### 2.1. Scale Development Process

This scale development study was conducted in May 2020 and followed the steps in the scale development and standardisation process [24] including (a) creating an initial pool of items and response scale, (b) assessing the content and face validity, and (c) testing the factor structure and reliability of the new scale.

#### 2.1.1. Scale Development and Generation of Items

The development of the scale initially began with a literature review that focused on the characteristics of e-learning experiences and their dimensions in higher education [8,18,24,25,26,27,28,29,30,31]. In addition, we searched for suitable items from empirical studies that met the following criteria: (a) they contained items to evaluate students’ e-learning experiences, and (b) they included both students and teachers as participants. After the literature review, 53 items were generated and reviewed by three team members, reflecting the dimensions of the e-learning experiences sought. To assess the extent to which each item was valued by the students, participants were asked to respond to each item on a 5-point Likert scale. There is much debate over how many response-points a scale should contain during scale development. After careful consideration, it was agreed to use the 5-point scoring scale with responses ranging from 1—strongly disagree to 5—strongly agree.

#### 2.1.2. Assessing the Content and Face Validity

A pilot study was then conducted focusing on the content validity of the scale. The content validity index (CVI) of the scale was assessed using the approach proposed by Lindell and Brandt [26]. Fifteen higher education teachers, experts in e-learning, were invited to review and rate the items generated in the first step. The experts were asked to rate the relevance and clarity of each item on a 4-point Likert scale: completely relevant (4), somewhat relevant (3), strongly in need of revision (2), and irrelevant (1). For clarity, a 3-point Likert scale was used: 1—not clear, 2—item needs some revision, and 3—very clear. The CVI of each item was calculated by dividing the number of experts who rated the item with 3 or 4 by the total number of experts [27]. According to Polit and Beck [27], acceptable CVI values should be at least 0.75, reflecting good content validity. In addition, the experts were asked to rate and critically review the items for comprehension and provide feedback to improve the form of each item. After some minor text corrections to ensure terminological appropriateness and to correct spelling errors, as well as the deletion of two problematic items that were both based on a low CVI index, the initial scale was prepared (Supplementary Materials File S1). The proposed scale was named the E-Learning Experience Evaluation Scale (3E-Scale) and was designed to measure the construct of students’ e-learning experience.

#### 2.1.3. Construct Validity Assessment and Reliability of the Scale

Confirmatory factor analysis was used to assess the scale’s construct validity [28]. The purpose of construct validity is to check whether the scale measures what we want it to measure, whether it provides exactly the data we want to obtain, and whether the variables are statistically significantly correlated with each other. The suitability of the data for factor analysis was checked using the Kaiser–Meyer–Olkin (KMO) index and Bartlett’s test of sphericity. The sample has a suitable size if the KMO index is at least 0.5. If Bartlett’s test of sphericity does not show the degree of statistical property (*p* < 0.05), that means the correlation matrix forms no statistically significant connections or the variables do not correlate with each other. In the data analysis, only those variables with an eigenvalue of communalities above 0.4 were considered appropriate [29]. 

The reliability of the 3E-Scale was assessed by analysing its internal consistency and stability based on the participants in the final step of this study. Cronbach’s alpha coefficient was used to evaluate the reliability and internal consistency of the scale for each factor. The Cronbach’s α cut-off value was set at 0.700, which is considered an acceptable value [27].

### 2.2. Measuring E-Learning Experience

#### Participants

The convenience sample consisted of undergraduate (Bologna first-cycle, *n* = 283) and postgraduate (Bologna second and third cycle, *n* = 59) health and allied health professions. Students were recruited for the study according to the following inclusion criteria: (a) students who participated in the e-learning process during the epidemic COVID-19, (b) students from the Faculty of Health Sciences at the University of Primorska. All students met the inclusion criteria at that time. The students reported spending 0 to 12 h per day on the computer (M = 4.06, SD = 2.382), 0 to 10 h per day on the computer for educational purposes (M = 3.43, SD = 1.879), and 0 to 6 h per day in the e-classroom platform (M = 1.81, SD = 1.372). Data were collected from 342 participants, of whom 61 were male and 281 were female. In 2020, 936 students were studying at the faculty. The calculated sample size based on 95% confidence level with 5% margin of error was 273. Although there is no universal agreement on the appropriate sample size for factor analysis, it should be noted that the minimum number is at least 100 or three times the number of all statements (3 × 51 = 153) [30]. The respondents’ age ranged from 19 to 49 years (M = 23.83, SD = 5.944). See Table 1 for a summary of the participants.

### 2.3. Data Collection

Participants were emailed a link to an online questionnaire. Consent to participate in the study was given by clicking on the embedded link and starting the survey. The online questionnaire was accessible through the open-source online survey application 1KA (https://www.1ka.si/d/en, accessed on 3 May 2020) in May 2020 and was accompanied by a statement of the purpose and method of completion. As part of the data collection process, 1KA supported the creation of a database that contained participants’ responses without their personal information to ensure anonymity. Participation in the survey was voluntary. During data analysis, data were accessible only to the lead researcher. After completion of the survey, all data collected were simultaneously transferred and exported to SPSS version 26.0 computer software (SPSS Inc., Chicago, IL, USA) for statistical analysis.

### 2.4. Data Analysis

To determine the 3E-Scale’s psychometric properties, the following statistical analyses were performed: descriptive statistics, Cronbach’s alpha coefficient to determine internal consistency, confirmatory factor analysis to estimate the factor structure, and Pearson’s correlation coefficient to determine the correlation between the variables. The normality of the data was then tested by the Shapiro–Wilk test. Since the data were not normally distributed (*p* < 0.0001), both the nonparametric Kruskal–Wallis H-test and the Mann–Whitney U-test were used to establish statistically significant differences between groups. A *p*-value ≤ 0.05 was considered significant.

### 2.5. Ethical Considerations

The principles established in the Declaration of Helsinki [31] were followed. The study was approved by the Commission for Scientific Research Work at the Faculty of Health Sciences, University of Primorska, on 24 April 2020 (No: 2420-21-20/2020). All data were treated confidentially. Informed consent was obtained from all participants who were willing to engage in the second step of the study to review the created items and complete the questionnaire in the third step.

## 3. Results

Confirmatory factor analysis (CFA) was performed to assess the scale’s validity. The analysis of the correlation between the variables showed their relevance, as confirmed by the Kaiser–Meyer–Olkin index and Bartlett’s test of sphericity (KMO = 0.954, Bartlett’s test of sphericity χ^2^ = 8409.632, df = 630, *p* = 0.000). These results indicate that the sample population was acceptable. Only those items whose eigenvalue of the communalities exceeded 0.4 were considered in the data analysis. Namely, items that did not share sufficient variance with another variable (14 out of 51 items) were excluded from the analysis (Supplementary Materials File S1). The factor weight matrix also showed that, apart from the items excluded, the factor loadings were higher than 0.40. The univariate normal distribution of the indicator variables was then determined by analysing the values of skewness and kurtosis. For all indicator variables, the values of skewness and kurtosis were 1 or less than 1, which is acceptable for performing CFA (−0.058–1.000) and means that there are no major deviations from the normal distribution.

The factor model explains 61% of the variance. The highest eigenvalue belongs to the first factor and is 15.479 and explains 42.999% of the total variance (the second factor 3.013 and 8.369%; the third factor 1.1896 and 5.266% and the fourth factor 1.642 and 4.560) (see Table 2). We named the factors: (1) “Effectiveness of learning”; (2) “Teacher’s role”; (3) “Delivery and support”; and (4) “Perceived barriers”. The first factor “Effectiveness of learning” included 14 items reflecting the usability of the e-learning platform through effectiveness of learning, opportunities for learning, learning needs, learning success, and flexible learning. The second factor “Teacher’s role” included 11 items reflecting teachers’ digital competencies. The third factor “Delivery and support” included 7 items reflecting the accessibility and variety of learning activities for different learning styles and the availability of teachers for tutoring and support. The last factor “Perceived barriers” included five items that reflected some difficulties students may encounter during the e-learning process.

The arithmetic means for the 3E-Scale factors or subscales range from 3.07 (“Effectiveness of learning”) to 3.68 (“Teacher’s role”). All mean values are higher than the median of the 3E-Scale, indicating a tendency to score positively on each subscale (although in the final statistical analysis, four items need to be reverse-coded). The standard deviations range from 0.74 (“Teacher’s role”) to 0.90 (“Effectiveness of learning”), indicating an adequate response variance.

The correlations of the coefficients between the latent variables are significant, ranging from 0.23 to 0.80 (with a median of 0.515). Among the three factors, moderate to high correlations (above 0.70) predominate, namely for “Effectiveness of learning”, “Teacher’s role” and “Delivery and support”, while very low correlations were found with the “Perceived barriers” factor (correlations ranging from 0.23 to 0.39). However, all correlations are statistically significant at *p* < 0.001 (Table 3).

The assessment of the 3E-Scale identified by the students are (M = 3.42, SD = 0.565 (95% CI 3.30, 3.42), *p* = 0.000). Reliability of the 3E-Scale was assessed using reliability and stability tests for all four scale factors. The results showed a high degree of reliability for the entire 3E-Scale (Cronbach α = 0.935), for both the “Effectiveness of learning” factor (Cronbach α = 0.952) and the “Teacher’s role” factor (Cronbach α = 0.920), as well as a good degree of reliability for the “Delivery and support” factor (Cronbach α = 0.887) and a weaker degree of reliability for the “Perceived barriers” factor (Cronbach α = 0.636) (Table 4). 

Finally, we calculated reliability using intraclass correlation, with values between 0.5 and 0.75 indicating moderate reliability [32]. We found an excellent value of 0.924 (95% CI 0.911, 0.936), *p* = 0.000) for the 3E-Scale, with subscale correlations ranging from 0.602 to 0.942.

To examine the relationship between participants in terms of their gender and study programme in relation to the 3E-Scale, the Mann–Whitney U test and the Kruskal–Wallis H test were performed (Table 5).

Female students have more positive experience with e-learning, although the results are not statistically significant (*p* > 0.05), with the exception of Factor 1—Effectiveness of learning, where the experience is not only rated better by female students, but the results show that there are statistically significant differences between the groups (*p* < 0.05). When comparing students’ experience with e-learning based on the study programme, the results show some differences between them. However, some statistically significant differences (*p* < 0.05) are shown in the evaluation of Factor 1—Effectiveness of learning, where nursing students evaluate it higher than students of other study programmes. In addition, nursing and dietetics students perceive more barriers related to the open-source learning management system than other students (*p* < 0.05). There are also no statistically significant differences between groups when comparing e-learning experiences by year of study (*p* > 0.05), with the exception of Factor 2—Teacher’s role, where first-year students rate the role of the teacher in e-learning higher than second- and third-year students (M = 3.91, SD = 0.750; M = 3.82, SD = 0.681; M = 3.55, SD = 0.681; *p* < 0.05, as follows).

## 4. Discussion

COVID-19 has particularly increased the trend towards e-learning worldwide, including in the field of medicine and caring professions [10]. This study aimed to develop, validate, and test the psychometric properties of a scale for evaluating students’ e-learning experiences among health and allied health professions students. It was focused on designing a measurement tool to help identify students’ experiences in the e-learning environment, thereby contributing to designing and advancing new learning/teaching approaches that meet learners’ needs. Students’ learning environments can be improved to achieve positive e-learning outcomes [22]. However, to be responsive to students’ needs, they must be monitored regularly.

The systems or e-platforms that provide educational opportunities are multidisciplinary in nature. Numerous researchers from different fields, such as computer science and information technologies, psychology, pedagogy, and educational technology use, have attempted to evaluate such systems in favour of e-learning. Some researchers have studied the technological components of e-learning systems, while others have looked only at the effect of the human factor of learning and teaching through these systems in terms of student and teacher satisfaction [9]. While these individual assessment frameworks offer some practical solutions, they meet the needs only to a limited extent and, most importantly, do not meet the needs of all participants in the pedagogical process [13,17,21,23]. The e-learning evaluation scale (3E-Scale) we developed includes four concepts to measure students’ perceived satisfaction with the pedagogical process within e-learning.

While the e-platform is an effective medium for teaching and learning in the current educational environment, there is a need for a deeper understanding of the drivers that motivate learners to engage with, adopt, and do well with e-learning. The scale we compiled includes several concepts from the fields of ICT and contemporary educational science. The obtained results based on KMO and Bartlett’s test of sphericity show that the implementation of CFA on the collected data was appropriate. Through factor analysis, which allows an explanation of the common variance of several variables at the same time, we obtained a four-factor model, namely: (1) Effectiveness of learning; (2) Teacher’s role; (3) Delivery and support; and (4) Perceived barriers. In the final version of the scale, a total of 14 items were excluded because they did not share sufficient variance with other items, reducing the original 51-item scale to 37 items.

The overall reliability and stability of the questionnaire and for individual factors proved to be very good. Although the level of reliability was slightly lower for the last set of questions relating to perceived barriers, it could be at the very limits of acceptability. The questionnaire’s validity was tested over several steps in order to examine the construct in more detail. The association between the factors ranged from moderate to high. We can therefore conclude that the psychometric properties of the 3E-Scale are very good and that the four-factor model is conceptually suitable to explain the e-learning experience at this stage of the study. 

Factor 1 of the model, “Effectiveness of learning”, combines items that assess the effectiveness of e-platforms for learners to acquire new knowledge. Even though the arithmetic mean for this subscale is not one of the highest (3.07), the study results confirm that students recognize e-learning as an effective teaching/learning method. As found in previous studies, e-learning increases educational effects by significantly improving the level of knowledge. This in turn requires teachers to monitor e-learning readiness and create/adapt an optimal learning environment to further support effective learning outcomes [5,22]. 

One of the most important roles within e-learning is that held by the teacher (Factor 2). Regardless, in our study, this did not prove to be the most important factor influencing user satisfaction within the pedagogical process. Kim, Kim, and Lee [22] similarly found that learning flow can be achieved through e-learning without teachers or facilitators. Despite this limitation of e-learning where teachers do not have direct control over students, pedagogical strategies use technology to ensure that the learning experience is engaging and interactive, provides opportunities for learner reflection, and promotes teacher presence and social and cognitive presence. An effective digital learning environment requires ongoing teacher support, prompt feedback on assignments, and responses to emails and questions in interactive forums [17,21,22]. Faculty support among nursing students has been shown to be related to persistence in nursing studies and academic success and to contribute to the development of resilience [13].

Learners are introduced to the digital learning platform to ensure they can use it effectively (Factor 3). The elements that can ultimately affect individual learning outcomes are the teacher’s attitude to the technology, their knowledge of its functionalities, their consideration of individual learning styles, and the use of methods and techniques suitable for e-learning. Although the teacher’s role in e-learning is different from his or her traditional role, a teacher’s technology literacy is a necessary skill because it affects learning outcomes, learner satisfaction, and the success of a facilitator’s role [12]. Furthermore, in terms of effective learning outcomes, the design of the digital learning platform must link teaching pedagogy to educational outcomes so that students master course content and improve their critical thinking, problem-solving, and communication skills [19].

The fact is that there are still many factors that represent certain barriers to learning while using e-platforms (Factor 4), such as familiarity with the system, its capabilities, various applications that the system offers, and the user’s attitude to the system [33], as was seen in the findings among female and male students. On the other hand, many other factors also hinder the adoption of e-learning innovations, such as financial difficulties associated with the acquisition of new technology, lack of motivation and need for adoption, or insufficiently qualified teaching staff [34]. It was apparent that nursing and dietetics students perceived many more barriers related to open-source learning than did students in allied health professions, as both of these study programmes include much more practical training that was not possible during the lockdown. This and many other reasons related to the sudden shift to e-learning have caused confusion among students (and teachers). Students’ anxiety is also an important factor in their learning mechanisms and their perceptions of the e-learning experience [21]. Therefore, future research attempts should investigate the negative effects of e-learning on students and possible interventions that teachers or facilitators can implement in the e-learning process.

### Limitations

This study has some limitations. Some of them are related to the methodological approach, others to the perception of personal experience and satisfaction, since these are highly subjective dimensions of each individual. One limitation to consider is the sampling approach, which did not focus sufficiently on representativeness. However, as this was the development and validation phase of the study, it was evaluated as sufficient in terms of the number of participants and in line with the current recommendations [35]. In the next stage, we propose to use stratified random sampling instead of convenience sampling to test other variables that emerge from the sample. In addition, the four-factor model showed satisfactory psychometric properties, but may not have covered the extent of students’ experiences with e-learning, as the items were intended to reflect within domains. Although our study suggests four factors, future studies should further refine the scale items and conduct a confirmatory factor analysis and test-retest analysis with new student samples. Although the scale has promising psychometric properties, there is another weakness expressed in the fourth factor. If the study were repeated with a new sample of students, more items could load on Factor 3, although our study suggests a four-factor model. Another limitation might be that the study was conducted only with Slovenian-speaking participants and the 3E-Scale might perform differently when translated into other languages worldwide. Further studies should test the 3E-Scale for cultural sensitivity and consequently perform a cross-cultural adaptation of the scale. However, a future validation study is recommended to enhance the strengths of its utility. 

## 5. Conclusions

Despite its many advantages, an e-platform-based educational process may contain some gaps that can seriously affect the results of this form of educational process. Therefore, in order to maintain the appropriate quality of the educational process, it is advisable that teachers carefully consider the content to be presented through the e-platform and the intended teaching approaches to be used before conducting an e-lesson. Currently, there is a lack of well-established instruments for use to evaluate the e-learning experience from the students’ perspective. At a time when the COVID-19 pandemic has created a worldwide need to adapt the implementation of the teaching process to the altered circumstances, our aim was to develop and adapt the measurement tool and to evaluate its psychometric properties given that sensitivity in this area is extremely high among students.

Our study confirmed that the tool we developed is effective in assessing students’ e-learning experience and, consequently, the quality of the pedagogical process based on e-learning satisfaction. This concept should not be neglected as it is related to students’ engagement in the educational process and effective ways to achieve learning outcomes. Even though the study was conducted among students, the scale could be useful at all levels of education in different disciplines where e-learning is used. However, further research would be needed for such application, including a mixed-methods approach that would more closely examine the phenomenon of e-learning satisfaction and the factors surrounding it. It should also be mentioned that this study is open to improvement in terms of incorporating new knowledge in the field of education as well as developments in ICT. This makes it necessary that future studies are conducted periodically and maintain the same research methodology, which is consistent with previous approaches and allows for the expansion and improvement of the tools used to determine the effectiveness of e-learning.

## Figures and Tables

**Table 1:** Demographic and other characteristics of the participants in step 3 (*n* = 342).

Characteristic	*n*	%
Gender		
Male	61	17.8
Female	281	82.2
Year of study		
1st year	160	46.8
2nd year	98	28.7
3rd year	84	24.5
Study programme		
Nursing	155	45.3
Physiotherapy	27	7.9
Nutritional counselling—dietetics	98	28.7
Kinesiology	62	18.1

**Table 2:** Confirmatory factor analysis results of the 4-factor solution with factor loadings (*n* = 342).

Variables		Factor Loadings	Mean Score (SD)	I-CVI (R, C)
Factor 1	It is easier to learn through the e-learning platform.	0.780	2.55 (1.163)	0.94
	I am sure that the eClassroom is a very effective learning tool.	0.771	3.36 (1.143)	0.94
	The e-learning platform provides an opportunity to learn more than with the traditional form of education.	0.766	2.58 (1.144)	1.0
	The eClassroom meets all my learning needs.	0.756	2.82 (1.238)	1.0
	eClassroom learning improves my learning success in the subject.	0.738	2.76 (1.069)	1.0
	E-learning provides a long-term opportunity to acquire new knowledge (new techniques, etc.) in an easy way.	0.730	3.51 (1.089)	1.0
	The eClassroom allows me to manage my learning time much more efficiently and complete the assigned tasks much more easily.	0.718	3.30 (1.236)	1.0
	The eClassroom helps me to approach learning in a more systematic way.	0.699	3.21 (1.081)	1.0
	The eClassroom has enabled me to familiarise myself with the subject matter.	0.693	3.09 (1.135)	1.0
	E-learning offers more flexible learning (more freedom than traditional learning).	0.689	3.41 (1.133)	1.0
	Overall, I find the eClassroom a successful learning environment.	0.677	3.52 (1.063)	0.94
	I enjoy participating in an organised course of studies in the eClassroom.	0.677	3.49 (1.159)	1.0
	The eClassroom makes it easier for me to communicate with the teacher and other students.	0.673	2.80 (1.165)	1.0
	The eClassroom as a learning environment improves my learning.	0.567	3.19 (1.074)	1.0
Factor 2	The teacher skilfully presents the learning contents through the eClassroom.	0.798	3.85 (0.873)	1.0
	The teacher addresses students’ problems and tries to find a suitable solution through communication in the eClassroom.	0.780	3.78 (0.973)	1.0
	The teacher responds quickly to students’ questions through emails provided by the eClassroom.	0.766	3.69 (0.992)	1.0
	The teacher has created a pleasant online learning environment in the eClassroom.	0.762	3.80 (0.888)	1.0
	The teacher is adept at communicating via the eClassroom.	0.750	3.75 (0.906)	1.0
	The teacher frequently updates lecture handouts and promptly fixes any errors that occur in the learning content available through the eClassroom.	0.745	3.80 (0.852)	1.0
	Communication with the instructor via the eClassroom is important and valuable.	0.615	3.80 (0.868)	1.0
	I am aware of the opportunity to send messages and communicate with the teacher and other students via the eClassroom.	0.592	3.86 (0.952)	1.0
	The teacher encourages us to communicate with other students through the interactive tools provided by the eClassroom.	0.583	3.22 (1.067)	1.0
	Communicating with the teacher through the eClassroom is not complicated.	0.532	3.68 (0.977)	1.0
	The teacher clearly presents the assessment criteria to the students through the eClassroom.	0.498	3.43 (1.003)	1.0
Factor 3	I can easily navigate through the eClassroom system.	0.826	3.59 (0.866)	1.0
	Navigating the eClassroom is very easy.	0.773	3.53 (0.944)	1.0
	I can find the information I need in the eClassroom.	0.771	3.68 (0.836)	1.0
	The software instructions and navigation in the eClassroom are very clear.	0.624	3.68 (0.846)	1.0
	The possibility of technical support in the eClassroom is available.	0.614	3.27 (0.915)	1.0
	The eClassroom supports interactivity between students and the system through chats, forums, discussions etc.	0.505	3.32 (1.005)	1.0
	The graphical user interface of the eClassroom is suitable for e-learning systems (icons, windows, other tools, etc.).	0.462	3.53 (0.888)	1.0
Factor 4	The e-learning platform cannot be used without proper guidance.	0.782	3.11 (1.087)	1.0
	An explanation of use and operation is required before using the e-learning platform.	0.753	3.59 (1.047)	1.0
	Use of the e-learning platform is time-consuming.	0.418	2.55 (1.065)	1.0
	E-learning is difficult from a technical and organisational point of view.	0.444	3.04 (1.178)	1.0
	The more experienced one is with using a computer, the more inclined one is to use e-learning.	0.426	3.20 (1.048)	1.0

Note. Accumulated total explained variance = 61%. Bartlett’s Test of Sphericity: χ^2^ = 8409,632, *p* < 0.0001; Kaiser–Meyer–Olkin value = 0.954; SD—Standard Deviation; Factor 1—Effectiveness of learning, Factor 2—Teacher’s role, Factor 3—Delivery and support, Factor 4—Perceived barriers; Factor rotation: Varimax with Kaiser normalisation; eClassroom—open-source learning management system-Moodle; Rating the scale is based on five answer alternatives, from 1—strongly disagree to 5—strongly agree; I-CVI (R, C)—Items Content Validity Index (Relevancy, Clarity).

**Table 3:** Correlation matrix for the factors of the 3E-Scale.

Factors/Subscales	1	2	3	4
Effectiveness of learning	-	0.68 **	0.71 **	−0.39 **
Teacher’s role	−0.68 **	-	0.80 **	−0.23 **
Delivery and support	0.71 **	0.80 **	-	0.32 **
Perceived barriers	−0.39 **	−0.23 **	0.32 **	-

Note. **—Correlation is significant at the 0.01 feature level.

**Table 4:** Reliability tests of the 3E-Scale scale and its four factors.

Factors/Subscales	*n*	M	SD	Cronbach α Coefficient	95% CI	*p* Value
Effectiveness of learning	14	3.21	0.891	0.952	3.02–3.21	<0.001
Teacher’s role	11	3.82	0.707	0.920	3.62–3.77	<0.001
Delivery and support	7	3.57	0.693	0.887	3.44–3.59	<0.001
Perceived barriers	5	3.20	0.692	0.636	3.02–3.17	<0.001
Entire 3E-Scale	37	3.42	0.565	0.935	3.30–3.42	<0.001

Note. SD—Standard Deviation.

**Table 5:** Characteristics of the sample population concerning the 3E-Scale and the four subscales.

Variable	Factor 1	Factor 2	Factor 3	Factor 4	3E-Scale
	M(SD)				
	Test Value/*p*				
Gender					
Male	2.79 (0.931)	3.64 (0.807)	3.57 (0.797)	3.00 (0.539)	3.27 (0.712)
Female	3.29 (0.881)	3.82 (0.685)	3.71 (0.670)	3.40 (0.521)	3.51 (0.632)
	(U)7034.00/ 0.028	(U)8098.00/ 0.499	(U)7772.00/ 0.269	(U)7677.00/ 0.199	(U)7258.00/ 0.061
Study programme					
Nursing	3.36 (0.876)	3.73 (0.649)	3.71 (0.665)	3.40 (0.516)	3.44 (0.624)
Physiotherapy	3.11 (0.787)	3.82 (0.545)	3.86 (0.424)	3.20 (0.481)	3.43 (0.443)
Nutritional counselling—dietetics	3.29 (0.892)	3.82 (0.745)	3.57 (0.704)	3.40 (0.548)	3.51 (0.681)
Kinesiology	2.79 (0.907)	3.82 (0.832)	3.57 (0.665)	3.00 (0.492)	3.35 (0.705)
	(χ^2^)14.245/ 0.003	(χ^2^)3.032/ 0.387	(χ^2^)5.631/ 0.131	(χ^2^)11.335/ 0.001	(χ^2^)6.221/ 0.101

Note. Factor 1—Effectiveness of learning, Factor 2—Teacher’s role, Factor 3—Delivery and support, Factor 4—Perceived barriers; Median (M); Standard deviation (SD); U value—Mann–Whitney U test; *χ^2^* value—Kruskal–Wallis H test; *p* value—statistical significance.

## Data Availability

The data are available from the corresponding author upon reasonable request as the participants were assured that it would remain confidential.

## Supplementary Materials

The following supporting information can be downloaded at: https://www.mdpi.com/article/10.3390/ijerph19084777/s1, Supplementary Materials File S1 title: rejected items.
