# Supplementary material for: Evaluation of E-Learning Experience among Health and Allied Health Professions Students during the COVID-19 Pandemic in Slovenia: An Instrument Development and Validation Study
Source: Int J Environ Res Public Health. 2022 Apr 14;19(8):4777. doi: 10.3390/ijerph19084777 (PMC9026893; doi:10.3390/ijerph19084777)
Supplement: Supplementary file 1 [file ijerph-19-04777-s001.zip › ijerph-1656126-supplementary.pdf]

Supplementary Materials File S1

Rejected items:

1. I learn best individually.
2. I prefer classical face-to-face teaching rather than individual learning.
3. E-learning is the most appropriate method to transfer knowledge.
4. Education via eClassroom gives a sense of isolation.
5. e-Learning is more suitable for people who prefer to learn individually.
6. The use of an eClassroom is more suitable for younger students.
7. The eClassroom must contain theoretical and practical aspects.
8. The eClassroom must allow participants to share ideas and discuss with others.
9. It is necessary to assess what has been learned after an e-learning course.
10. I am self-disciplined and it is easy for me to take time to read and do homework.
11. Overall, I am satisfied with eClassroom.
12. I have not found any system errors with eClassroom.
13. I am aware of all course announcements in eClassroom by using the 'Announcements' tool.
14. The fonts (style, color and saturation) are easy to read both on screen and in the printed version.
15. When I log in, I prefer eClassroom to provide me with a personalized entry page (e.g., with an indication of my progress, an indication of chapters I still need to revise, etc.).
16. I am more confident to learn through the eClassroom.
